# Numerical investigation of cracking behaviors in rock with interrupted double cross fractures under Brazilian splitting

**DOI:** 10.1038/s41598-025-26101-1

**Published:** 2025-11-26

**Authors:** Donglin Fan, Peidong He, Sushe Chen, Xinhua Zhao, Xin Zou, Yang Wu

**Affiliations:** 1 CHN Energy Shendong Coal Group Co., Ltd., Yulin, 719315 China; 2 State Key Laboratory of Water Resource Protection and Utilization in Coal Mining, National Institute of Low Carbon and Clean Energy, Beijing, 102209 China

**Keywords:** Engineering, Natural hazards, Solid Earth sciences

## Abstract

The stability of underground engineering in coal mines is influenced by the tensile properties of fractured rock masses. In this study, the finite element method with embedded zero-thickness cohesive elements (FEM-CZM) was employed to conduct numerical simulations of Brazilian splitting tests on specimens containing double intersecting pre-existing fractures, and the influence of fracture parameters on crack propagation characteristics was analyzed. The results indicate that the tensile stress zones are primarily distributed around the pre-existing fractures, while the compressive stress zones are mainly located at the central top and bottom of the specimen. The tips of the pre-existing fractures serve as stress concentration zones, and tensile failure is the primary cause of crack initiation. With the increase in the rock bridge inclination angle and main fracture inclination angle, both the number of cracks and the crack area show an upward trend. As the rock bridge length increases, the number of crack initiations and the crack area initially decrease slightly and then increase significantly. As the angle between main and secondary fractures increases, the number and density of cracks increase. The research findings provide a more scientific theoretical basis and design guidance for engineering stability assessment and disaster prevention.

## Introduction

In the field of mining engineering, accidents such as rock burst, coal and gas outburst, and roof water inrush often occur due to local tensile fracture of rock masses, leading to unstable propagation of micro-cracks and large-scale fractures, which ultimately trigger mining disasters^1–5^. These engineering disasters severely constrain the exploitation and utilization of resources, primarily due to an insufficient understanding of rock mass engineering materials. Studying the damage and strength characteristics of fractured rock masses is the scientific foundation for solving these engineering disaster challenges.

In recent years, numerous scholars have conducted extensive research on the mechanical properties of fractured rock masses and achieved significant results. Du et al.^6^ conducted uniaxial compression tests on sandstone with fractures, investigating the influence of different rock bridge quantities on the mechanical properties and failure characteristics of sandstone. Feng et al.^7^ performed uniaxial compression tests on specimens with two pre-existing fractures, studying the effects of rock bridge length and inclination angle on the mechanical properties of rocks. Qin et al.^8^ conducted uniaxial compression tests on cast specimens with different types of fractures, examining the influence of inclination angle, connectivity rate, aperture, and spacing on the mechanical properties and damage evolution of the specimens. Wang et al.^9^ carried out uniaxial compression tests on granite specimens with different fracture inclination angles to better explore the impact of discontinuous fractures on the mechanical properties and deformation characteristics of rock masses. Luo et al.^10^ conducted numerical simulations on rock masses containing various types of fractures, studying the influence of different fracture shapes and distributions on the failure behavior of rock masses. Zhang et al.^11^ performed uniaxial compression tests on rock specimens with fractures of different inclination angles, investigating the effects of fracture angles on crack evolution and fracture characteristics. Liu et al.^12^ conducted uniaxial compression tests on fractured rock masses, studying the influence of loading rates on their mechanical properties. However, existing research has predominantly focused on the mechanical behavior of fractured rock masses under uniaxial compression, with relatively fewer studies on the tensile properties of fractured rocks. Cross-fractured rock masses are widely distributed in slopes, underground engineering, and rock excavation areas, and their tensile properties are of significant importance in engineering practice, particularly in rock mass stability and failure control, where tensile strength is often a critical factor. Therefore, in-depth research on the tensile properties of fractured rocks not only helps to improve the theoretical system of rock mechanics but also provides a more reliable design basis for engineering practice.

Compared to laboratory experiments, numerical simulations offer advantages such as high precision in micro-scale calculations, intuitive representation, and cost-effectiveness, which can compensate for the limitations of laboratory experiments and allow for the observation of rock mass damage and fracture processes at a microscopic level^13–17^. Currently, most scholars tend to use discrete element software to study the mechanical properties and fracture evolution of fractured rock masses, while the Finite Element-Cohesive Zone Model (FEM-CZM) method is less frequently used. This method considers the composition and distribution of rock masses, providing a better description of the continuity and discontinuity characteristics of rock materials and effectively simulating the mechanical and failure behavior of rock masses^18–21^. This method considers the composition and distribution of rock masses, providing a better description of the continuity and discontinuity characteristics of rock materials and effectively simulating the mechanical and failure behavior of rock masses^18–20^. Du et al.^21^ investigated the tensile behavior of Brazilian disc specimens containing cracks based on the FEM-CZM method and investigated the influence of the distribution characteristics of crack holes on the tensile fracture behavior of the specimens. Han et al.^22^ used the FEM-CZM method to conduct shear numerical simulations on fractured rock masses, analyzing their mechanical properties and failure modes. Wu et al.^23^ employed the FEM-CZM method to study the shear behavior and crack evolution of rock masses with intermittent joints. Wang et al.^24^ used the global embedding of zero-thickness cohesive elements to simulate the shear process of jointed rock masses under constant normal load. These studies demonstrate that the FEM-CZM method has high accuracy in simulating the mechanical behavior and crack evolution of fractured rock masses, making it feasible to apply this method to simulate the tensile properties of fractured rock masses.

In summary, this paper adopts the FEM-CZM method to construct a numerical model of rock specimens with double-cross fractures, fully considering the composition and distribution characteristics of rock masses, which can better describe the continuity and discontinuity behavior of rock materials. Based on the FEM-CZM method, this paper investigates the mechanical response of rock masses with double-cross fractures in Brazilian splitting tests through numerical simulations, further revealing the tensile properties and deformation failure mechanisms of rock masses. The research results can provide theoretical references and a basis for the prevention and control of mining disasters.

## Modeling process

### Traction separation criterion

**Fig. 1:**
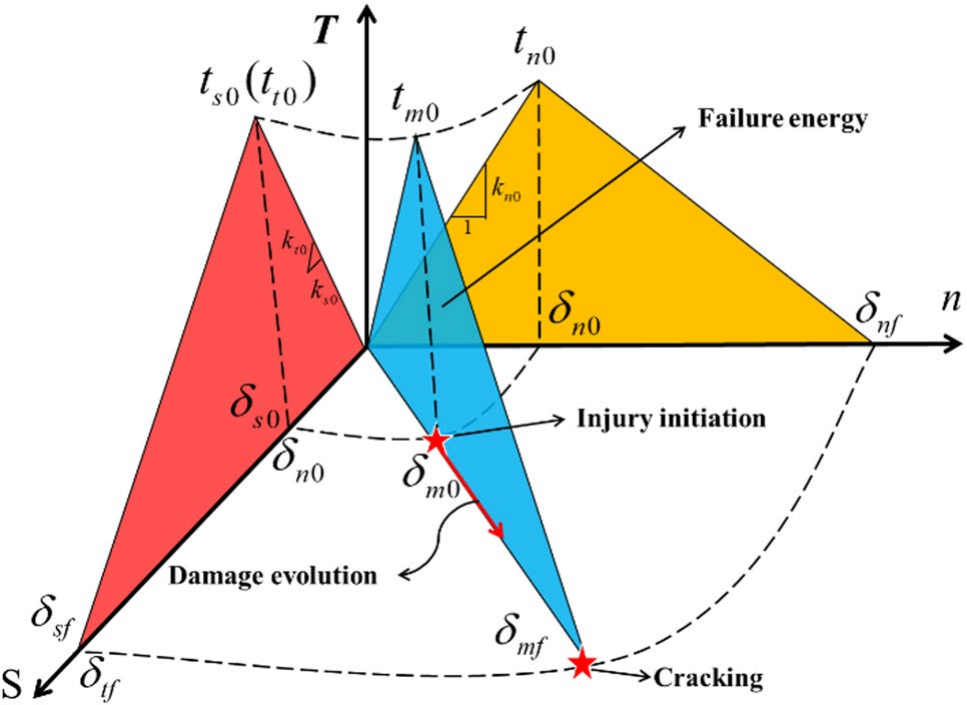
Traction separation criterion.

In the ABAQUS software, the cohesive zone model follows the traction-separation criterion, and its constitutive model is illustrated in Fig. 1. Specifically, the model exhibits linear elastic hardening before the damage reaches the evolution stage, and linear softening after the damage enters the evolution stage. By introducing a correction parameter to characterize the functional relationship between the external force applied to the cohesive element and the initial damage stress, the model becomes more suitable for analyzing elastoplastic problems. Before reaching the damage development stage, the external force applied to the cohesive element has not yet reached the initial damage stress, and the relationship between stress and strain follows Hooke’s law. The linear elastic behavior can be described by a matrix as follows:

1 $$t=\left\{ {\begin{array}{*{20}{c}} {{t_n}} \\ {{t_s}} \\ {{t_t}} \end{array}} \right\}=\frac{I}{L}\left[ {\begin{array}{*{20}{c}} {{E_{nn}}}&{{E_{ns}}}&{{E_{nt}}} \\ {{E_{ns}}}&{{E_{ss}}}&{{E_{st}}} \\ {{E_{nt}}}&{{E_{st}}}&{{E_{tt}}} \end{array}} \right]\left\{ {\begin{array}{*{20}{c}} {{\delta _n}} \\ {{\delta _s}} \\ {{\delta _t}} \end{array}} \right\}=\left[ {\begin{array}{*{20}{c}} {{K_{nn}}}&{{K_{ns}}}&{{K_{nt}}} \\ {{K_{sn}}}&{{K_{ss}}}&{{K_{st}}} \\ {{K_{nt}}}&{{K_{st}}}&{{K_{tt}}} \end{array}} \right]\left\{ {\begin{array}{*{20}{c}} {{\delta _n}} \\ {{\delta _s}} \\ {{\delta _t}} \end{array}} \right\}$$

In the equation, *t* represents the traction stress vector; *t*_n_ is the normal traction force; *t*_s_ and *t*_t_ are the two tangential traction forces; *δ*_n_、*δ*_s,_ and *δ*_t_ are the three components of strain; *L* is the initial thickness of the cohesive element; and *K* is the stiffness matrix of the cohesive element.

After reaching the damage evolution stage, the stiffness of the cohesive element will decrease linearly. In ABAQUS, the scalar *D* is used to represent the degree of damage. The initial value of the damage scalar *D* is set to 0 and gradually increases from 0 to 1. To accurately describe the damage evolution of the cohesive element, a function related to the effective displacement *δ*_m_ is introduced as follows:

2 $${\delta _m}=\sqrt {{\delta _n}^{2}+\delta _{s}^{2}+\delta _{t}^{2}}$$

Where 〈*δ*_m_〉 represents the Macaulay bracket, ensuring that the cohesive element only undergoes damage under tensile loading. When 〈*δ*_m_〉≥0, it indicates tension, and 〈*δ*_n_〉 equals *δ*_n_. When 〈*δ*_n_〉≤0, it indicates compression, and 〈*δ*_n_〉 equals zero.

The damage variable *D* evolves according to the following expression:

3 $$D=\frac{{{\delta _{mf}}({\delta _{mm}} - {\delta _{mo}})}}{{{\delta _{mm}}({\delta _{mf}} - {\delta _{mo}})}}$$

Where: *δ*_mm_ is the maximum effective displacement in the loading history; *δ*_mo_ is the effective displacement at the onset of crack initiation; *δ*_*mf*_ is the effective displacement at failure.

During the softening stage, the normal stress *t*_n_ is expressed as:

4 $$\begin{array}{*{20}{c}} {{t_n}=\left\{ {\begin{array}{*{20}{c}} {(1 - D){t_{no}},}&{{t_{no}} \geqslant 0} \\ {{t_{no}},}&{{t_{no}}<0} \end{array}} \right.} \\ {{t_s}=(1 - D){t_{so}}} \\ {{t_t}=(1 - D){t_{to}}} \end{array}$$

The stiffness of the cohesive element can be expressed by the following equation:

5 $$\begin{gathered} {K_n}=(1 - D){K_{no}} \hfill \\ {K_s}=(1 - D){K_{so}} \hfill \\ {K_t}=(1 - D){K_{to}} \hfill \\ \end{gathered}$$

The separation displacement change is represented as:

6 $$\begin{gathered} {\delta _n}=\frac{{{t_n}}}{{(1 - D)}}{K_n} \hfill \\ {\delta _s}=\frac{{{t_s}}}{{(1 - D)}}{K_s} \hfill \\ {\delta _t}=\frac{{{t_t}}}{{(1 - D)}}{K_t} \hfill \\ \end{gathered}$$

### Generation process of zero-thickness cohesive elements

**Fig. 2:**
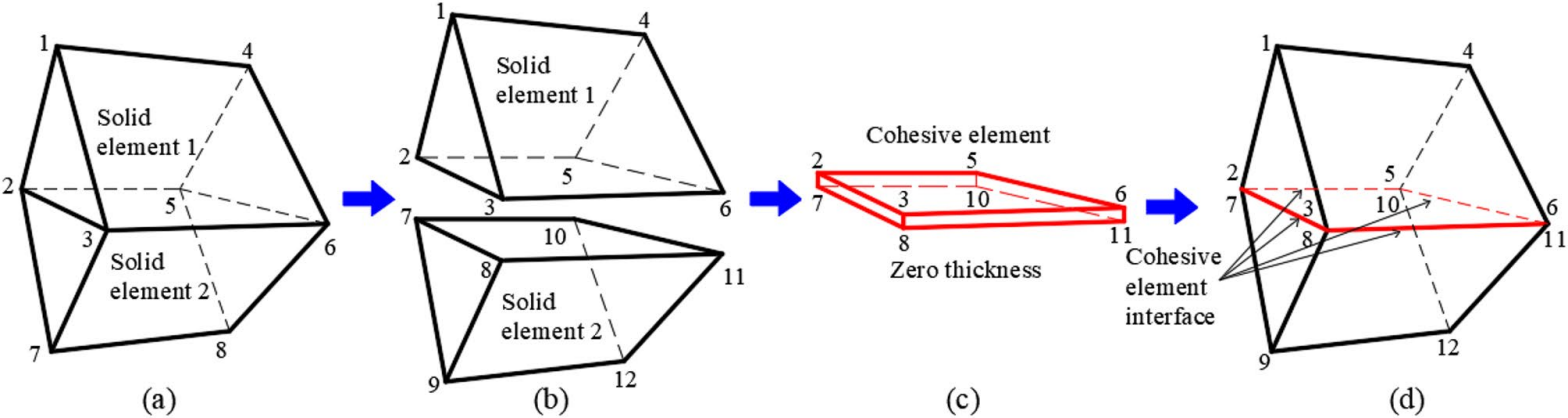
Flowchart of zero-thickness cohesive element generation: (**a**) Two adjacent solid elements; (**b**) Rearranging the nodes of the solid elements; (**c**) Zero-thickness cohesive elements; (**d**) Insertion of cohesive elements.

To more accurately simulate the discontinuity between real rock particles and achieve the macroscopic crack initiation and failure behavior of rock masses, this study employs the method of embedding zero-thickness cohesive elements within pre-existing cracks in the initial finite element mesh. The process of embedding cohesive elements is illustrated in Fig. 2. As shown in Fig. 2a, the solid elements are first discretized, and the node information is read. In Fig. 2b, the finite element mesh and nodes on the coincident surfaces are separated, and the nodes are rearranged. In Fig. 2c, zero-thickness cohesive elements are embedded between the originally coincident element faces. These cohesive elements have zero thickness, ensuring that the model dimensions remain unchanged after embedding, and the cohesive elements share nodes with the adjacent mesh. Finally, Fig. 2d shows the zero-thickness cohesive element model.

### Model building

**Fig. 3:**
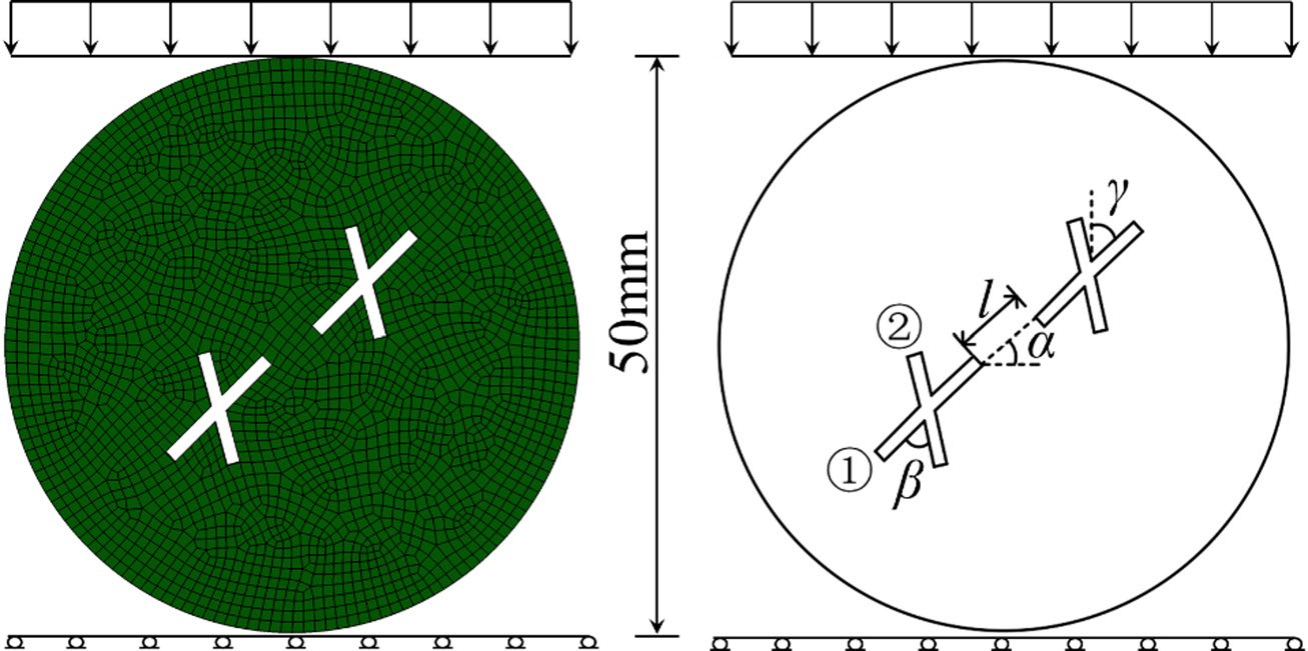
Model of the specimen with double cross fractures.

The Brazilian splitting specimen model containing double pre-existing cross fractures is shown in Fig. 3. It should be noted that when the rock mass fractures, solid elements may collide and displace relative to each other. Therefore, this study employs global contact to define the contact behavior. Global contact can only be applied to three-dimensional surface contact, so the influence of the direction perpendicular to the loading surface on the splitting behavior can be neglected. The specimen dimensions are determined based on indoor Brazilian splitting tests. In the simulation, the specimen used for the Brazilian splitting test is a standard sample with a diameter of 50 mm. The specimen contains double pre-existing cross fractures, with the main fracture length being 12 mm, the secondary fracture length being 10 mm, and the fracture width being 1.2 mm. For the boundary conditions, a constant normal stress is applied to the upper surface of the specimen at a loading rate of 0.1 mm/s to ensure quasi-static loading. The lateral boundaries of the model are assigned a friction coefficient of 0.3. It is worth noting that the opening area of each specimen remains constant to eliminate the influence of the opening ratio (opening area/specimen area). The mechanical parameters used in the simulation are based on the research of Du et al.^21^, and the numerical parameters are listed in Table 1.

**Table 1:** Mesoscopic parameters of the numerical model.

Materials	Parameters	Unit	Value
Rock-like materials	Density	kg·m^-3^	2500
	Young’s modulus	GPa	15
	Poisson’s ratio	/	0.3
Cohesive elements	*K*_*n0*_	GPa·mm^-1^	15
	*K*_*s0*_	GPa·mm^-1^	5.28
	*K*_*t0*_	GPa·mm^-1^	5.28
	*t*_*n*_	MPa	5.5
	*t*_*s*_	MPa	20
	*t*_*t*_	MPa	20
	Model-I fracture energy	N/mm^-1^	0.055
	Model-II fracture energy	N/mm^-1^	0.16

To investigate the fracture characteristics and crack propagation behavior of rock containing intermittent double cross joints, this study conducts Brazilian splitting simulations on specimens with two intermittent pre-existing cross fractures, as shown in Fig. 3. The specimen has a diameter of 50 mm, with the main fracture (Fracture 1) length of 12 mm, the secondary fracture (Fracture 2) length of 10 mm, and both fracture widths of 1.2 mm. The rock Bridge length is denoted as *l*, the rock Bridge inclination angle as *α*, the main fracture inclination angle as *β*, and the angle between the main and secondary fractures as *γ*. Here, the rock Bridge inclination angle *α* is the angle between the rock Bridge and the horizontal direction, and the main fracture inclination angle *β* is the angle between the main fracture and the vertical direction. The mesoscopic parameters of the specimen with intermittent double cross fractures are listed in Table 1. to study the influence of the geometric distribution of double cross fractures on the tensile strength and crack propagation characteristics of brittle rock, the following four simulation schemes are designed (Table 2):

**Table 2:** Test scheme.

Simulation scheme	Variable parameters	Fixed parameters
Variation in rock bridge inclination angle (*α*)	*α* = 45°, 60°, 75°	*l* = 5 mm,*β* = 45°,*γ* = 60°
Variation in rock bridge length (*l*)	*l* = 2.5 mm, 5 mm, 7.5 mm	*α* = 45°,*β* = 45°,*γ* = 60°
Variation in main fracture inclination angle (*β*)	*β* = 45°, 60°, 75°	*l* = 5 mm,*α* = 45°,*γ* = 60°
Variation in angle between main and secondary fractures (*γ*)	*γ* = 60°, 75°, 90°	*l* = 5 mm,*α* = *β* = 45°

These simulation schemes aim to systematically explore the effects of different geometric parameters on the mechanical behavior and fracture patterns of rock specimens with double cross fractures.

## Simulation results

### Principle of crack generation

**Fig. 4:**
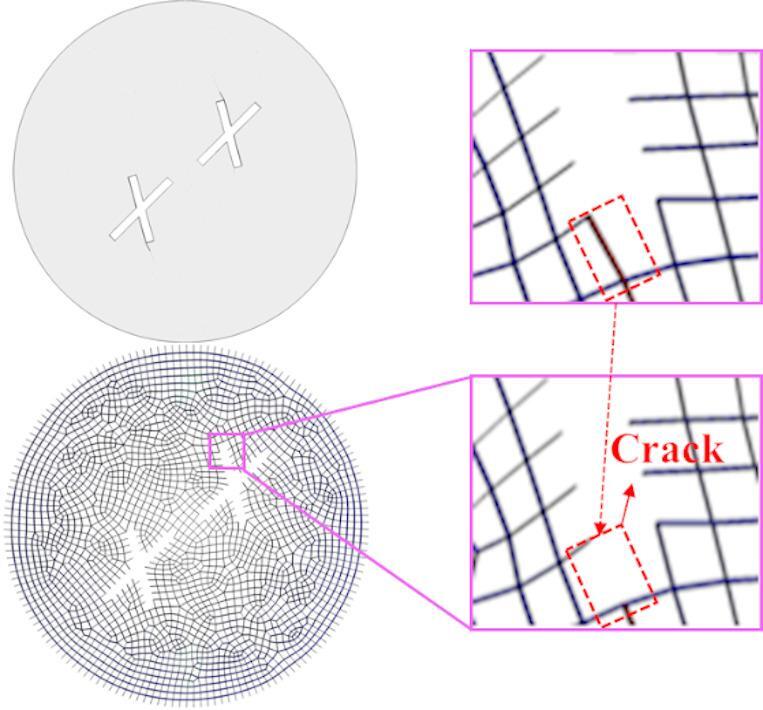
Principle of crack initiation.

This section discusses the influence of different parameter variations on the mechanical properties of specimens containing double cross fractures. Figure 4 illustrates the cracking mechanism of the model specimen. The initiation of cracks originates from the failure of cohesive elements. In the early stages of the test, the specimen is subjected to a small load, and the cohesive elements remain intact, with no cracks appearing in the specimen. As the load increases, the cohesive elements at the upper end of the secondary fracture are the first to enter a state of failure, leading to irreversible damage and a subsequent decrease in tensile strength. The formation of local cracks and the reduction in stiffness cause the primary tensile resistance zone to shift to other locations, resulting in a more uniform stress distribution. This process not only enhances the tensile strength of the specimen but also triggers the generation of cracks.

### Crack initiation mechanism

**Fig. 5:**
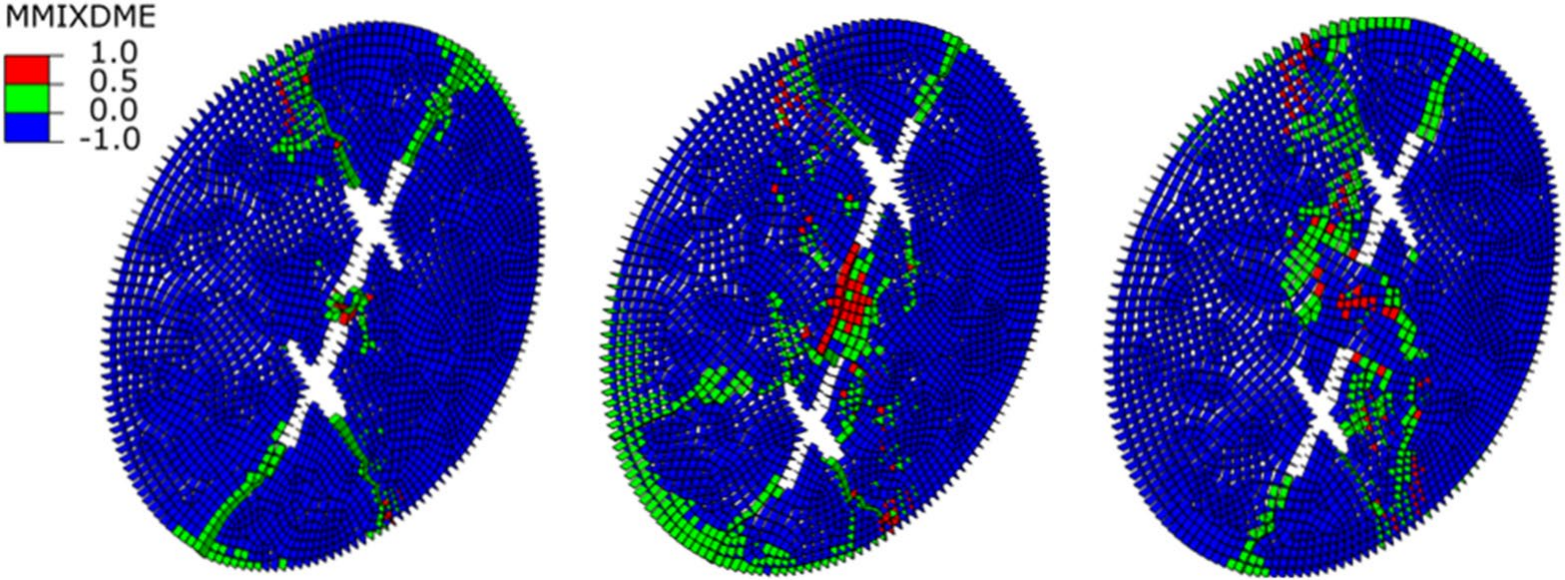
Crack initiation mechanism.

To explore the mechanism of crack initiation, this study identifies the types of cracks generated based on the damaged cohesive elements. The MMIXDME parameter, which represents the proportion of fracture modes during the damage evolution process, is used to determine the damage type of the cohesive elements. Specifically, when the MMIXDME value is in the range of 0 to 0.5, the cohesive elements are primarily subjected to tensile damage, resulting in tensile cracks. When the MMIXDME value is at -1, no damage occurs to the cohesive force unit. When the MMIXDME value is in the range of 0.5 to 1, the cohesive elements are dominated by shear damage (shear cracks). When the MMIXDME value reaches 1, the cohesive elements are subjected to shear but do not undergo further damage. Based on the above criteria, the mechanism of crack initiation is illustrated in Fig. 5. Tensile cracks are generated at the tips of both the main and secondary fractures of the two intersecting fractures, and these tensile cracks extend to the edges of the specimen, forming through-going cracks. In the rock bridge region of the specimen, both tensile cracks and shear cracks are generated.

### Analysis of mechanical properties in Brazilian splitting

#### Influence of rock bridge inclination angle on crack characteristics

**Fig. 6:**
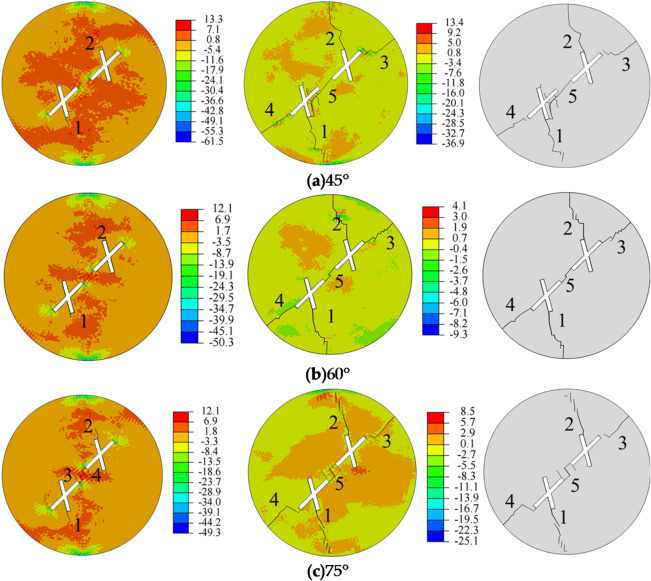
Stress evolution and crack propagation under different rock bridge inclination angles.

Figure 6 shows the influence of the rock bridge inclination angle on crack propagation and the final failure mode of the specimen. Observations during the crack initiation stage reveal that when the rock bridge inclination angle is 45° and 60°, crack initiation starts at the tips of the secondary fractures (points 1 and 2) of the two intersecting fractures, with crack initiation angles of 195° and 155°, respectively. When the rock bridge inclination angle is 75°, crack initiation occurs at two locations: near the tips of the main fractures (points 3 and 4) close to the center of the specimen and near the tips of the secondary fractures (points 1 and 2) close to the specimen boundary, with crack initiation angles of 115° and 160°, respectively. It is evident that as the rock bridge angle increases, the number of crack initiation points also increases. The increase in the rock bridge inclination angle alters the spatial configuration between pre-existing fractures, thereby significantly regulating the stress field distribution around them. As the inclination angle increases, the fractures transition from being relatively independent to approximately parallel, enhancing their synergistic stress disturbance effects. This induces notable tensile stress concentration, particularly within the rock bridge region. Consequently, both the tips of the main and secondary fractures become potential sources for tensile cracking, which macroscopically manifests as an increase in the number of crack initiation sites. From the stress distribution, it can be observed that the tensile stress zones are primarily concentrated around the pre-existing fractures, while the compressive stress zones are mainly located at the central top and bottom of the specimen. The tips of the pre-existing fractures serve as stress concentration zones, and tensile failure is the primary cause of crack formation.

Regarding crack propagation, the sequence and starting points of crack initiation, as well as the crack propagation paths, are largely similar. The crack patterns in the specimen exhibit an X-shape. When the rock bridge inclination angle is 45°, the five cracks are straight. When the rock bridge inclination angle is 60° and 75°, the five cracks are zigzag-shaped. As the stress increases, the generated cracks continue to propagate and intersect, leading the specimen into the crack coalescence stage. It can be observed that when the rock bridge inclination angle is 45° and 75°, the cracks at the main fracture tips (points 3 and 4) do not fully connect. When the rock bridge inclination angle is 60°, the cracks at the main fracture tips (points 3 and 4) fully connect. The cracks at the secondary fracture tips (points 1 and 2) do not fully connect for any of the three rock bridge inclination angles. These cracks connect with the pre-existing fractures, ultimately leading to specimen failure. To explore the debonding mechanism of the cracks, it is necessary to identify the types of cracks generated based on the damaged cohesive elements. The MMIXDME parameter, which represents the proportion of fracture modes during the damage evolution process, is used to determine the damage type of the cohesive elements. Specifically, when the MMIXDME value is in the range of 0 to 0.5, the cohesive elements are primarily subjected to tensile damage, resulting in tensile cracks. When the MMIXDME value is in the range of 0.5 to 1, the cohesive elements are dominated by shear damage. When the MMIXDME value is -1, it indicates that the cohesive elements are not damaged at that stage.

**Fig. 7:**
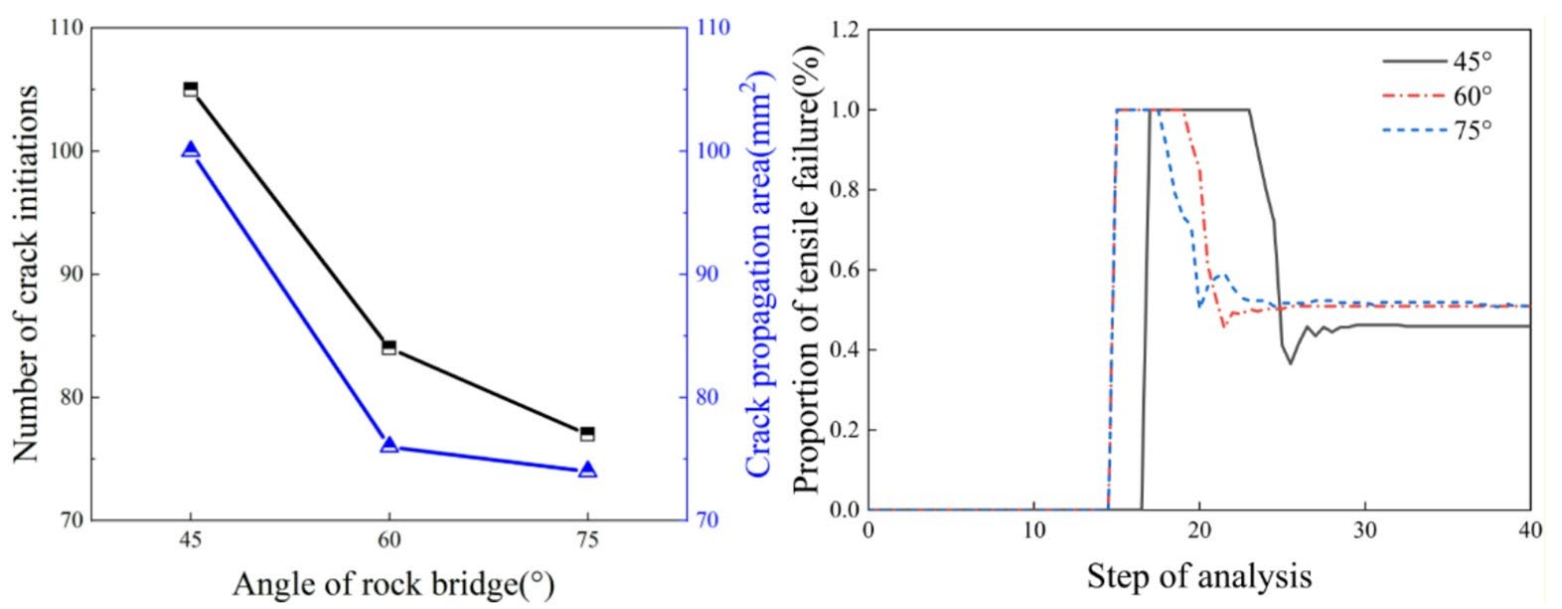
Crack parameters for different rock bridge inclination angles.

From the analysis of Fig. 7, it can be observed that as the rock bridge angle increases, both the number of crack initiations and the crack area show a decreasing trend. The number of crack initiations is the highest when the rock bridge angle is 45°, and it decreases as the rock bridge angle increases. When the rock bridge angle increases from 45° to 60°, the number of crack initiations decreases from 105 to 84, representing the largest reduction. When the rock bridge angle is 45°, the crack area reaches its maximum value of 100.25. When the rock bridge angle increases from 45° to 60°, the crack area decreases by 24. When the rock bridge angle is 60° and 75°, the crack areas are similar, measuring 75.53 and 76.51, respectively. Tensile failure is the primary cause of crack initiation. As the splitting process continues, the proportion of tensile failure gradually decreases and stabilizes at around 60% when the specimen fails. As the rock bridge angle increases, the proportion of tensile failure at the time of specimen failure becomes progressively higher.

#### Influence of rock bridge length on crack characteristics

**Fig. 8:**
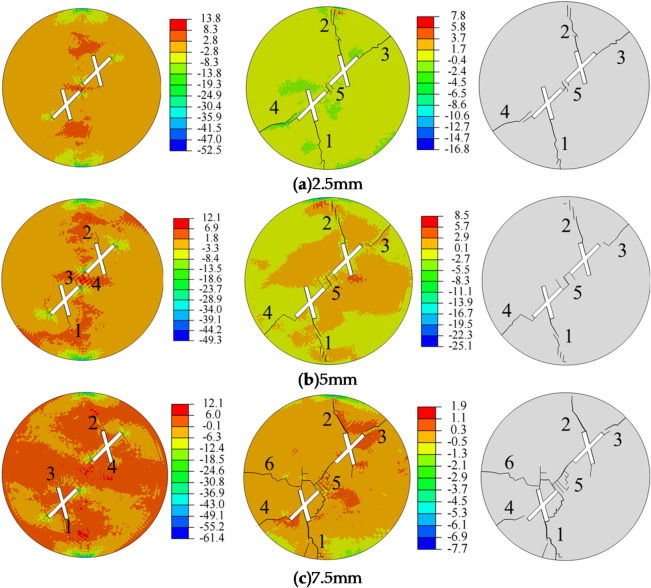
Stress evolution and crack propagation under different rock bridge lengths.

Figure 8 illustrates the influence of the rock bridge length on crack propagation and the final failure mode of the specimen. Observations during the crack initiation stage reveal the following: When the rock bridge length is 2.5 mm, no significant cracks appear in the specimen. When the rock bridge length is 5 mm, crack initiation starts at the tips of the main fractures (points 3 and 4) and the tips of the secondary fractures (points 1 and 2) of the two intersecting fractures. The crack initiation angles at the main fracture tips (points 3 and 4) are both 120°, while the crack initiation angles at the secondary fracture tips (points 1 and 2) are 160° and 150°, respectively. When the rock bridge length is 7.5 mm, crack initiation occurs at the tips of the secondary fractures (points 1 and 2) of the two intersecting fractures, with crack initiation angles of 175° and 210° at points 1 and 2, and 185° and 170° at points 3 and 4, respectively. From the stress distribution, it can be observed that the tensile stress zones are primarily concentrated around the pre-existing fractures, while the compressive stress zones are mainly located at the central top and bottom of the specimen. The tips of the pre-existing fractures serve as stress concentration zones, and tensile failure is the primary cause of crack formation. As the rock bridge length increases, the tensile stress zones in the specimen also expand significantly, and spalling phenomena occur in the tensile regions.

Regarding crack propagation, when the rock bridge length is 2.5 mm and 5 mm, the sequence and starting points of crack initiation, as well as the crack propagation paths, are largely similar. The crack patterns in the specimen exhibit an X-shape. When the rock bridge length increases to 5 mm, the five cracks in the specimen become larger. When the rock bridge length increases to 7.5 mm, the five cracks become more pronounced, and spalling phenomena occur. Additionally, new cracks form at other locations, such as point 6. It is evident that as the rock bridge length increases, the number and density of cracks in the specimen also increase. As the stress increases, the generated cracks continue to propagate and intersect, leading the specimen into the crack coalescence stage. It can be observed that when the rock bridge length is 2.5 mm and 5 mm, the cracks at the main and secondary fracture tips do not fully connect. When the rock bridge length is 7.5 mm, the cracks at the main fracture tips (points 3 and 4) do not fully connect, while the cracks at the secondary fracture tips (points 1 and 2) fully connect. These cracks connect with the pre-existing fractures, ultimately leading to specimen failure.

**Fig. 9:**
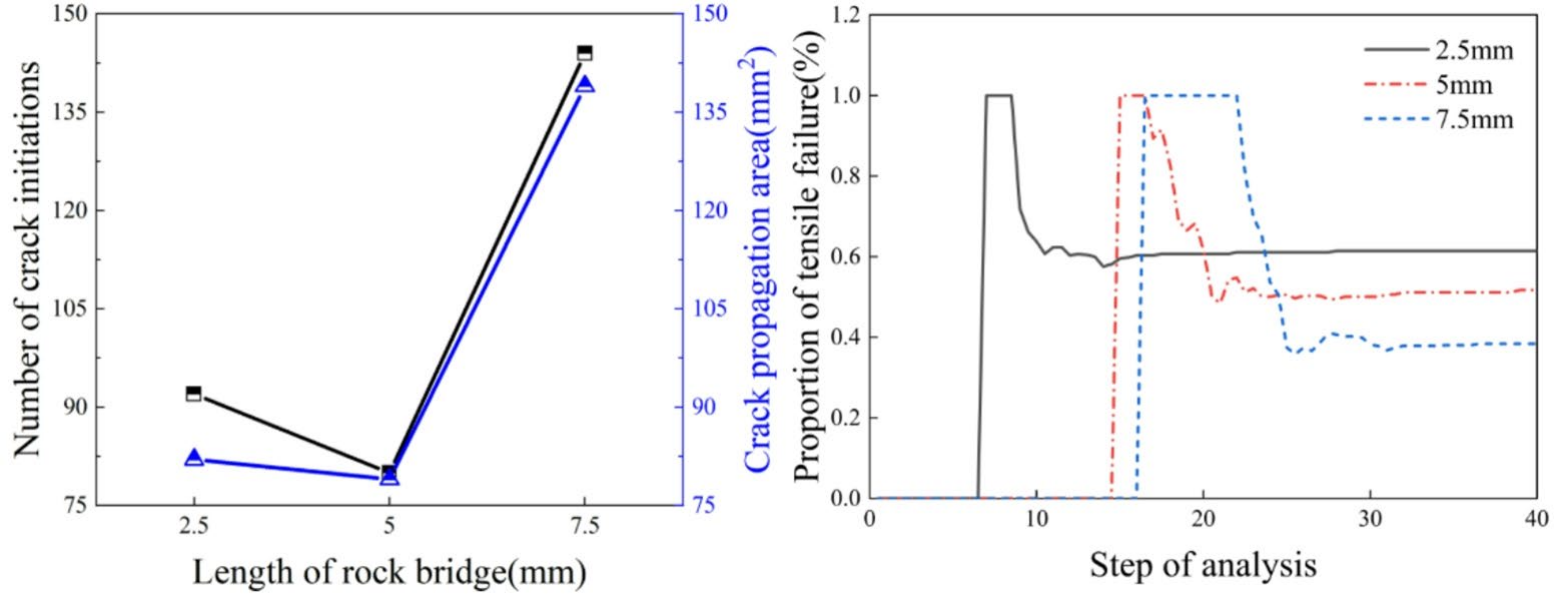
Crack parameters for different rock bridge lengths.

From the analysis of Fig. 9, it can be observed that as the rock bridge length increases, the number of crack initiations and the crack area show a trend of initially decreasing slightly and then increasing significantly. When the rock bridge length increases from 2.5 mm to 7.5 mm, the number of crack initiations increases by 52, the crack area increases by 56.32, and the crack volume decreases by 70. When the rock bridge length increases from 5 mm to 7.5 mm, the number of crack initiations shows the largest increase, rising from 80 to 144, with an increase rate of approximately 40%. Simultaneously, the crack area also experiences the largest increase, rising from 78.74 to 138.58. Tensile failure is the primary cause of crack initiation. As the splitting process continues, the specimen generates both tensile cracks and shear cracks, and the proportion of tensile failure gradually decreases, stabilizing when the specimen fails. As the rock bridge length increases, the proportion of tensile failure at the time of specimen failure gradually decreases. When the rock bridge lengths are 2.5 mm, 5 mm, and 7.5 mm, the proportions of tensile failure are 60%, 50%, and 40%, respectively.

#### Influence of main fracture inclination angle on crack characteristics

**Fig. 10:**
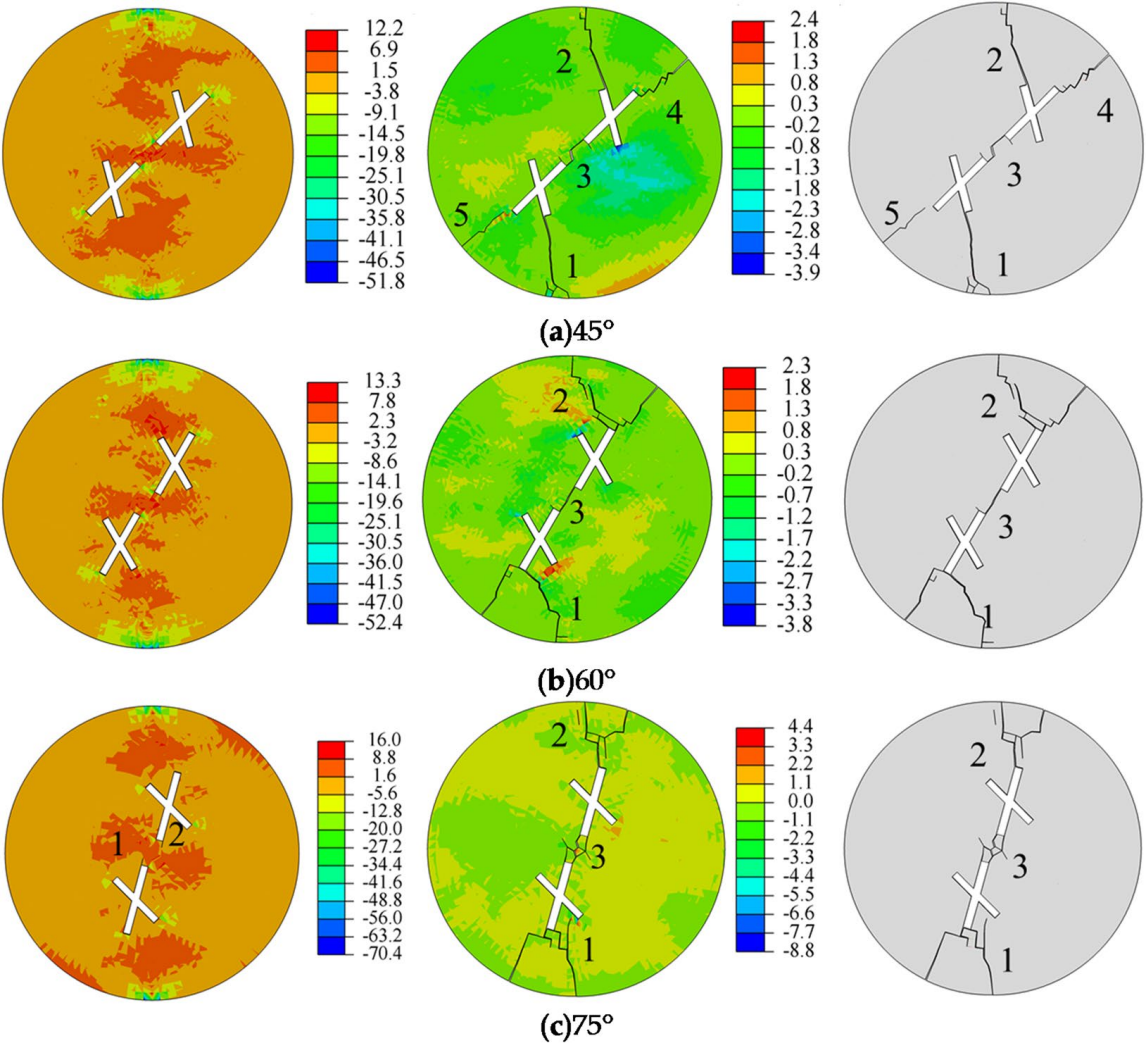
Stress evolution and crack propagation under different main fracture inclination angles.

Figure 10 shows the influence of the main fracture inclination angle on crack propagation and the final failure mode of the specimen. Observations during the crack initiation stage reveal that when the main fracture inclination angle is 45° or 60°, no significant cracks appear in the specimen. When the main fracture inclination angle is 75°, crack initiation starts at the tips of the main fractures (points 1 and 2) of the two intersecting fractures, with a crack initiation angle of 180°. From the stress distribution, it can be observed that the tensile stress zones are primarily concentrated around the pre-existing fractures, while the compressive stress zones are mainly located at the central top and bottom of the specimen. The tips of the pre-existing fractures serve as stress concentration zones, and tensile failure is the primary cause of crack formation.

Regarding crack propagation, the sequence and starting points of crack initiation, as well as the crack propagation paths, are largely similar. The crack patterns in the specimen exhibit an X-shape. When the main fracture inclination angle is 45°, cracks form at the tips of both the main and secondary fractures of the two intersecting fractures. However, as the main fracture inclination angle increases, cracks no longer form at the secondary fractures and only appear at the main fractures. Additionally, when the main fracture inclination angle is 75°, spalling phenomena occur at the center of the specimen. As the stress increases, the generated cracks continue to propagate and intersect, leading the specimen into the crack coalescence stage. It can be observed that when the main fracture inclination angle is 45°, the cracks at the main fracture tips (points 4 and 5) do not fully connect. When the main fracture inclination angle is 60° and 75°, the cracks at the main fracture tips (points 1 and 2) fully connect. These cracks connect with the pre-existing fractures, ultimately leading to specimen failure. Notably, as the main fracture inclination angle increases, the angle between the cracks decreases.

**Fig. 11:**
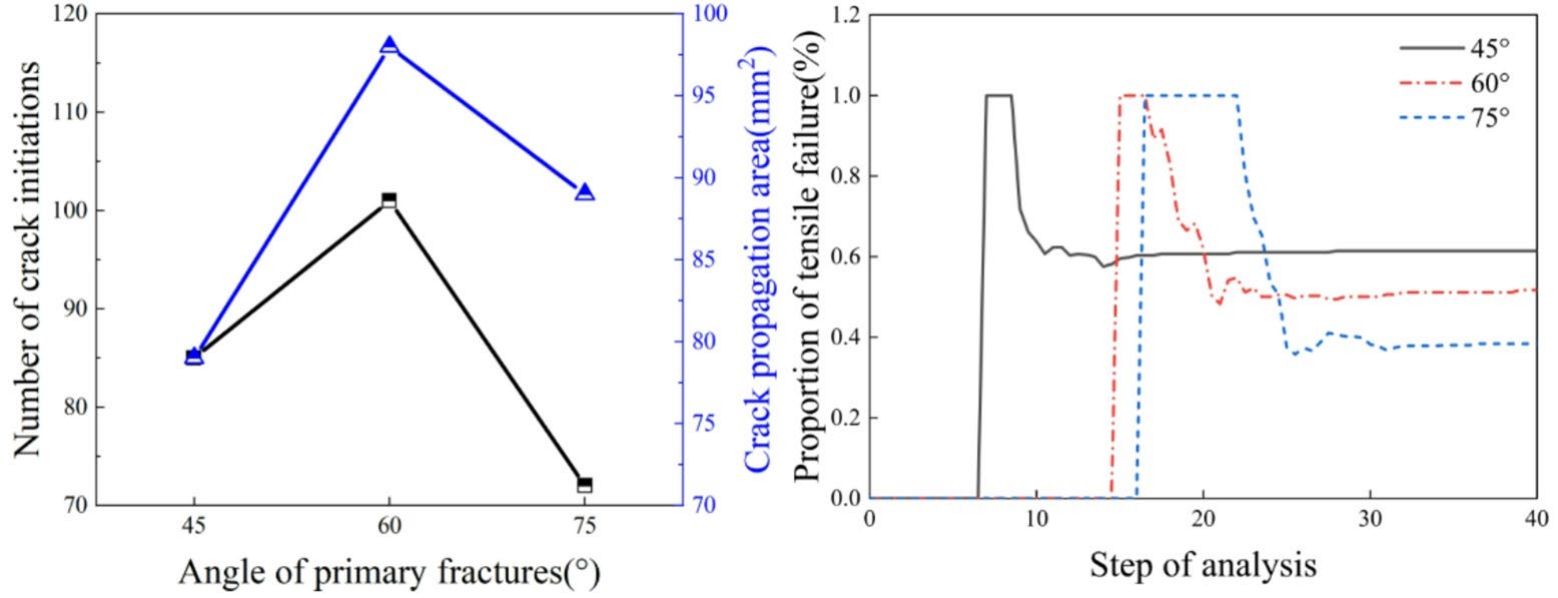
Crack parameters for different main fracture inclination angles.

From the analysis of Fig. 11, it can be observed that as the main fracture inclination angle increases, the number of crack initiations and the crack area show a trend of initially decreasing and then increasing. When the main fracture inclination angle increases from 45° to 60°, the number of crack initiations increases by 16. When the main fracture inclination angle increases from 60° to 75°, the number of crack initiations decreases by 29. When the main fracture inclination angle increases from 45° to 60°, the crack area increases by 14.59. When the main fracture inclination angle increases from 60° to 75°, the crack area decreases by 4.84. Tensile failure is the primary cause of crack initiation. As the splitting process continues, the specimen generates both tensile cracks and shear cracks, and the proportion of tensile failure gradually decreases, stabilizing when the specimen fails. When the main fracture inclination angles are 45°, 60°, and 75°, the proportions of tensile failure are 50%, 60%, and 45%, respectively.

#### Influence of fracture intersection angle on crack characteristics

**Fig. 12:**
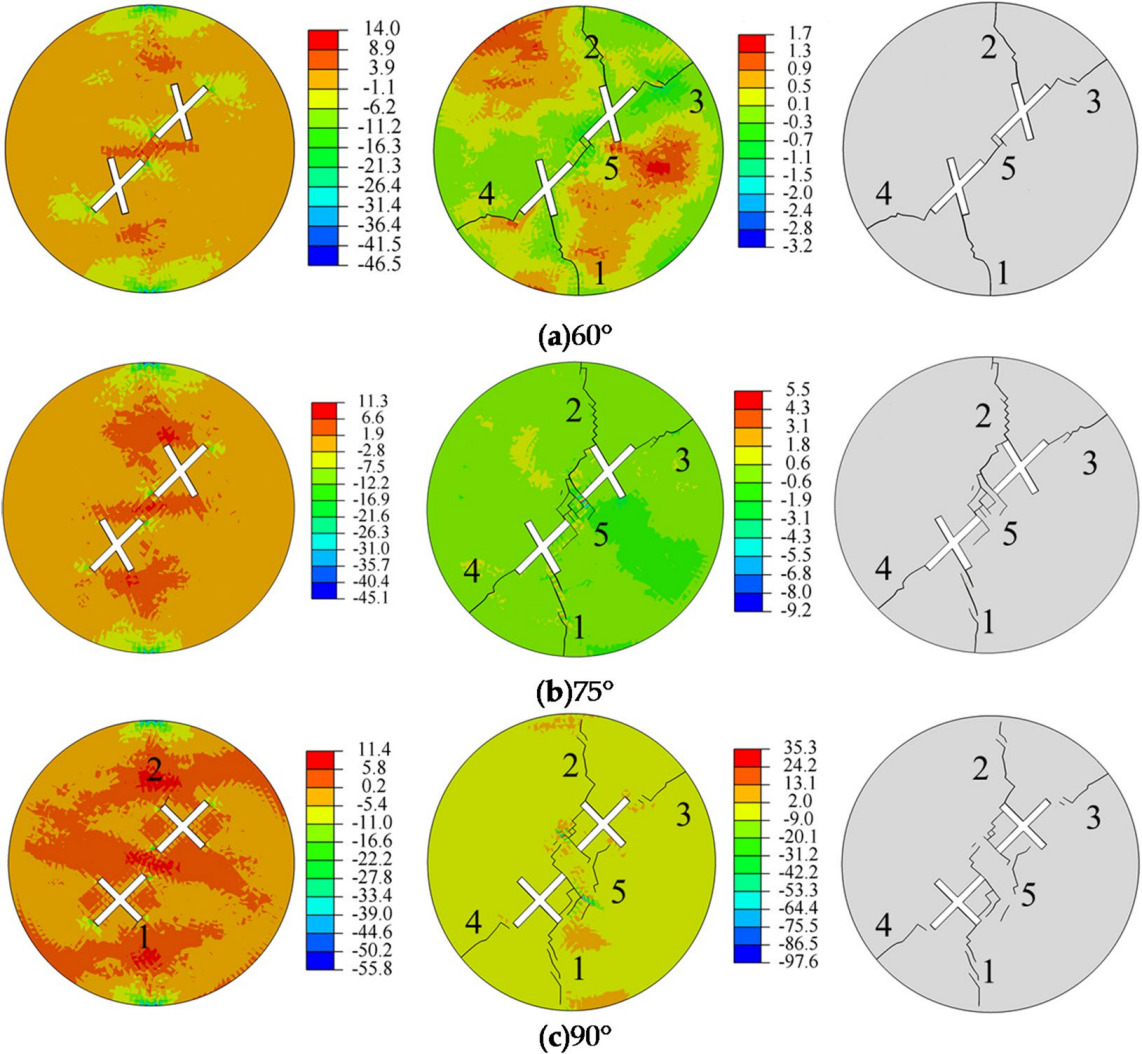
Stress evolution and crack propagation under different fracture intersection angles.

Figure 12 shows the influence of the fracture intersection angle on crack propagation and the final failure mode of the specimen. Observations during the crack initiation stage reveal that when the cross-fracture intersection angle is 60° and 75°, no significant cracks appear in the specimen. When the cross-fracture intersection angle is 90°, crack initiation starts at the tips of the secondary fractures (points 1 and 2) of the intersecting fractures. From the stress distribution, it can be observed that the tensile stress zones are primarily concentrated around the pre-existing fractures, while the compressive stress zones are mainly located at the central top and bottom of the specimen. The tips of the pre-existing fractures serve as stress concentration zones, and tensile failure is the primary cause of crack formation. Regarding crack propagation, the sequence and starting points of crack initiation, as well as the crack propagation paths, are largely similar. The crack patterns in the specimen exhibit an X-shape.

When the cross-fracture intersection angle is 60°, the five cracks are straight. When the cross-fracture intersection angle is 75° and 90°, the five cracks are irregular and exhibit spalling phenomena. It is evident that as the cross-fracture intersection angle increases, the number and density of cracks in the specimen also increase. As the stress increases, the generated cracks continue to propagate and intersect, leading the specimen into the crack coalescence stage. It can be observed that when the cross-fracture intersection angle is 60° and 90°, the cracks at the main fracture tips (points 3 and 4) do not fully connect. When the cross-fracture intersection angle is 75°, the cracks at the main fracture tips (points 3 and 4) fully connect. When the cross-fracture intersection angle is 75° and 90°, the cracks at the secondary fracture tips (points 1 and 2) do not fully connect. When the cross-fracture intersection angle is 60°, the cracks at the secondary fracture tips (points 1 and 2) fully connect. These cracks connect with the pre-existing fractures, ultimately leading to specimen failure.

**Fig. 13:**
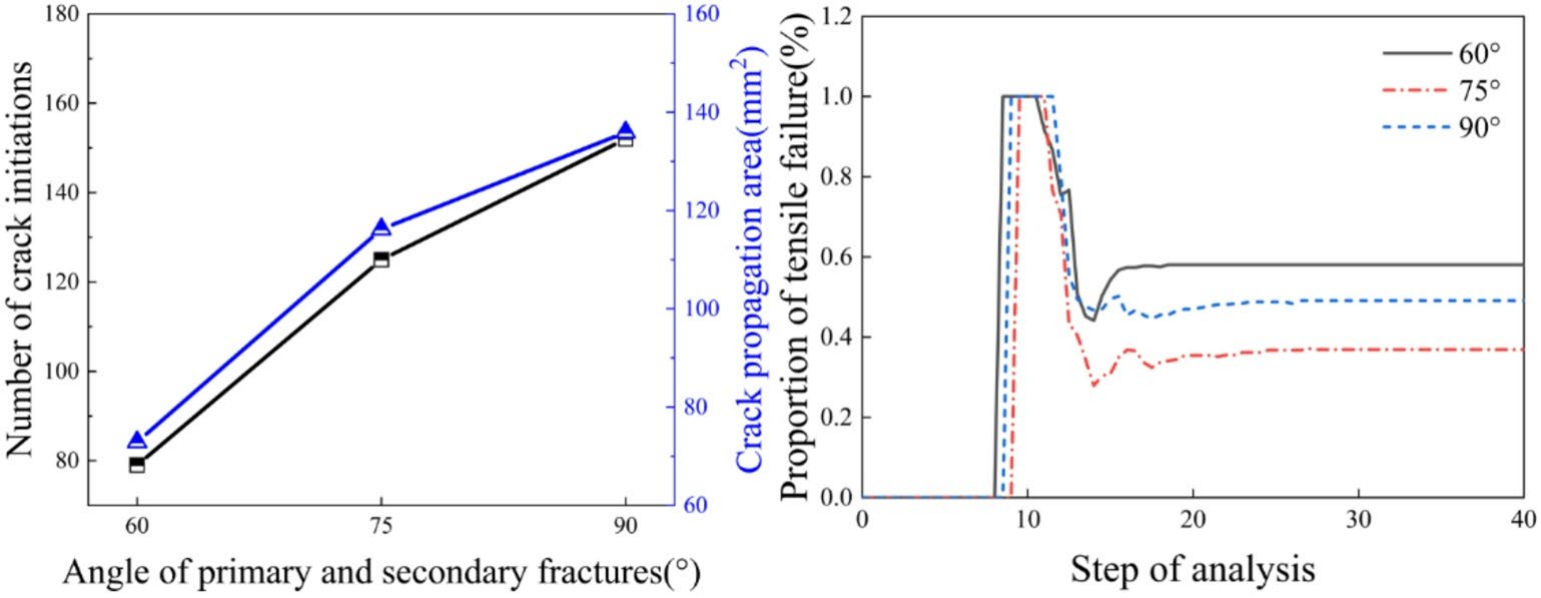
Crack parameters for different angles between the main and secondary fractures.

From the analysis of Fig. 13, it can be observed that as the angle between the main and secondary fractures increases, both the number of crack initiations and the crack area show an upward trend. When the angle between the main and secondary fractures increases from 60° to 90°, the number of crack initiations increases from 79 to 152, representing an increase of 73. The crack area increases from 72.88 to 135.90, rep-resenting an increase of 43.32. Tensile failure is the primary cause of crack initiation. As the splitting process continues, the specimen generates both tensile cracks and shear cracks, and the proportion of tensile failure gradually decreases, stabilizing when the specimen fails. When the angles between the main and secondary fractures are 60°, 75°, and 90°, the proportions of tensile failure are 60%, 40%, and 50%, respectively.

## Conclusions

This study, based on the FEM-CZM method, conducted numerical simulations of Brazilian splitting tests on rock masses containing double cross fractures, further analyzing the tensile properties and deformation failure mechanisms of the rock mass. Based on this method, the following main conclusions can be drawn:

1. From the stress distribution, it can be observed that the tensile stress zones are primarily concentrated around the pre-existing fractures, while the compressive stress zones are mainly located at the central top and bottom of the specimen. The tips of the pre-existing fractures serve as stress concentration zones, and tensile failure is the primary cause of crack formation. Additionally, the cracks generated after specimen failure mostly exhibit an X-shape.
2. The splitting process generally consists of four typical stages: compaction stage, elastic stage, yield stage, and failure stage. Crack initiation usually begins during the elastic stage. As the rock bridge angle increases, the number of crack initiation points also increases. As the rock bridge length increases, the number and density of cracks in the specimen also increase. Notably, as the angle between the cross fractures increases, the number and density of cracks in the specimen also increase.
3. Through the statistical analysis of the number of crack initiations and the crack area, it is evident that as the rock bridge angle and the angle between the main fractures increase, both the number of crack initiations and the crack area show an upward trend. As the rock bridge length increases, the number of crack initiations and the crack area initially decrease slightly and then increase significantly. Additionally, it can be observed that changes in the main fracture inclination angle have little impact on these two parameters.
4. Tensile failure is the primary cause of crack initiation. As the splitting process continues, the specimen generates both tensile cracks and shear cracks. Throughout the splitting process, the proportion of tensile failure remains relatively high.

## Data Availability

The datasets used and analyzed during the current study are available from the corresponding author on reasonable request.
